# Manipulation of drugs to achieve the required dose is intrinsic to paediatric practice but is not supported by guidelines or evidence

**DOI:** 10.1186/1471-2431-13-81

**Published:** 2013-05-21

**Authors:** Roberta H Richey, Utpal U Shah, Matthew Peak, Jean V Craig, James L Ford, Catrin E Barker, Anthony J Nunn, Mark A Turner

**Affiliations:** 1 Alder Hey Children’s NHS Foundation Trust, Eaton Road, Liverpool L12 2AP, UK; 2 Cheshire, Merseyside & North Wales Medicines for Children Local Research Network, Eaton Road, Liverpool, L12 2AP, UK; 3 School of Pharmacy & Biomolecular Sciences, Liverpool John Moores University, Byrom Street, Liverpool, L3 3AF, UK; 4 Norwich Medical School, Faculty of Medicine and Health Sciences, University of East Anglia, Norwich, NR4 7TJ, UK; 5 NIHR Medicines for Children Research Network Co-ordinating Centre, Institute of Child Health, University of Liverpool, Liverpool L12 2AP, UK; 6 Liverpool Women’s Hospital NHS Foundation Trust, Crown Street, Liverpool L8 7SS, UK

**Keywords:** Drug manipulation, Survey, Dosage forms, Children's medicines

## Abstract

**Background:**

A lack of age-appropriate formulations can make it difficult to administer medicines to children. A manipulation of the dosage form may be required to achieve the required dose. This study aimed to describe medicines that are manipulated to achieve the required dose in paediatric practice.

**Method:**

A structured, undisguised observational study and postal survey. The observational study investigated drug manipulations occurring in clinical practice across three sites. The questionnaire, administered to a sample of paediatric nurses throughout the UK, surveyed manipulations conducted and nurses’ experiences and views.

**Results:**

The observational study identified 310 manipulations, of which 62% involved tablets, 21% were intravenous drugs and 10% were sachets. Of the 54 observed manipulations 40 involved tablets with 65% of the tablets being cut and 30% dispersed to obtain a smaller dose. 188 manipulations were reported by questionnaire respondents, of these 46% involved tablets, 12% were intravenous drugs, and 12% were nebuliser solutions. Manipulations were predominantly, but not exclusively, identified in specialist clinical areas with more highly dependent patients. Questionnaire respondents were concerned about the accuracy of the dose achieved following manipulations and the lack of practice guidance.

**Conclusion:**

Manipulations to achieve the required dose occur throughout paediatric in-patient settings. The impact of manipulations on the efficacy of the drugs, the accuracy of the dose and any adverse effects on patients is not known. There is a need to develop evidence-based guidance for manipulations of medicines in children.

## Background

A lack of commercially-available, age-appropriate formulations makes it difficult to administer medication to children accurately [1-4]. Many medicines given to children use dosage forms designed for adults [5]. The magnitude of doses required throughout childhood can vary up to 100-fold [6]. A proportion of the dose in the available marketed dosage form may be required [7]. Medicines are thus manipulated by the physical alteration of a dosage form with the aim of achieving the required (usually smaller) dose for administration. Examples include splitting a tablet and administering a proportion or the further dilution of an injection when the available concentrations do not permit the paediatric dose to be measured accurately without dilution. Although commonly acknowledged among professionals as a widespread practice, reports about manipulations with the aim of achieving the required dose are limited. Manipulations may be time-consuming, can be inaccurate, and have unknown effects on the stability and bioavailability of the drug [3,8,9]. This risks the administration of toxic or sub-therapeutic doses. Drug manipulations may also increase the risk of errors. Dose calculation errors are the most common medication error in neonatal and paediatric practice [10]. Tablet manipulations have been encouraged (for adults) in some countries because of economic considerations. There may be little cost difference between different tablet strengths of the same drug [11,12]. Therefore it may be significantly cheaper to split tablets than buy the tablet with the optimal dose. A systematic review completed in this area [13] identified only one study (involving suppositories) that was not related to splitting tablets. Studies in the systematic review included drugs which are used in paediatric practice, though there were only two papers that included paediatric participants. Further research and practice guidance is needed.

A key barrier to good practice and research is the lack of understanding about the scope of manipulations in paediatric practice. There are few reports about the settings in which manipulations occur or the types of manipulations that are performed. Accordingly the aim of this study was to scope which dosage forms and drugs are routinely manipulated in paediatric practice. This study also investigated reasons for undertaking manipulations and concerns raised by those undertaking manipulations.

## Methods

### Design

In view of the lack of extant data about the scope of manipulations in paediatric practice we elected to gather data in two ways. Firstly we conducted a structured, undisguised, observational study of drug manipulations occurring in paediatric practice. Purposive sampling was used to identify the manipulations occurring in general and specialised clinical in-patient areas. Subsequently, a questionnaire was sent to paediatric nurses throughout the UK. The questionnaire was designed to provide complementary information about the nature of manipulations in a broader sample and to elicit the views of paediatric nurses about manipulations.

Table 1 provides the working definitions for dosage form manipulations that were used for this study.

**Table 1:** Definitions of manipulations for each dosage form

**Drug dosage form**	**Manipulation for dose accuracy includes**
Tablet	• split/broken/cut and a segment given
	• crushed and a proportion of the powder given
	• dispersed in liquid and a portion of the liquid given
Capsule	• opened, dispersed in liquid and a proportion of the liquid given
	• opened and a portion of the powder given
Sachet (powder)	• opened, dispersed in liquid and a portion of the liquid given
	• opened and a proportion of the powder given
Oral liquid	• diluted and a proportion given (to make the measurement of a small dose volume easier)
Suppository	• cut/split and a segment given
Nebuliser solution	• portion given
	• diluted and a proportion given
Enema/bladder irrigation	• proportion of sachet/unit given (the remainder then discarded)
	• portion of contents removed and the remainder given
Transdermal patch	• patch cut and a portion applied
	• portion of patch uncovered and applied
Intravenous injection	• reconstituted or ready prepared solution, further diluted to allow a smaller dose to be measured,
	• volume of fluid removed from IV container, drug added (to obtain accurate concentration for infusion)
	• drug added to infusion bag, portion with smaller dose removed and infused

### Observational study

The observational study was conducted at three sites; a large regional children’s hospital (where all 18 in-patient wards were included in the observational study); a regional specialist neonatal unit (54 cots), and a district general hospital with one paediatric (30 beds, two high dependency) and one neonatal ward (16 cots). The sites included 21 different in-patient areas and therefore simultaneous observations using a simple cross-sectional design were not feasible. Observations were conducted in blocks of two weeks. Each block was dedicated to a ward, or small number of wards at a particular site. During each two-week block potential manipulations for observation were identified prospectively via daily prescription reviews. This was supplemented by the use of alert cards which nurses were asked to complete where they had identified a manipulation while administering medicines. Where manipulations were identified, attempts were made to observe the manipulation occurring in practice. As manipulations have not been previously observed in practice a structured observational tool was devised. This tool was reviewed by clinical and research experts and piloted within five clinical areas prior to use in the study.

### Questionnaire study

The results of the observational study were used in the design of a questionnaire. This questionnaire was administered to paediatric nurses at a variety of geographical locations in the UK to reduce any potential local bias from observations undertaken at hospitals within one region. The questionnaire enabled the collection of additional data on the nature and type of drug manipulations in neonatal and paediatric practice and the clinical inpatient areas in which they occur. Additionally, respondents were asked to identify the reason that the manipulation occurred and there were questions asking about the availability of local documentation, any reference sources used prior to undertaking a manipulation and opportunity was given to describe any concerns or additional comments. The questionnaires were piloted with paediatric clinical and research nurses and a small number of changes made to ensure clarity for potential questionnaire respondents. All drug manipulations reported in the questionnaire responses were reviewed by an experienced paediatric clinical pharmacist (AJN) to ensure that they met the criteria to be considered a manipulation to achieve the required dose.

Paediatric nurse managers across the UK were contacted and requested to distribute the questionnaires to their staff. There was no direct communication between the research team and questionnaire respondents, all questionnaire responses were anonymous. Managers from 30 hospitals agreed to participate; relative to the size of their unit, they were sent questionnaires by post, with the option to request further quantities if required.

The closed questions within the questionnaire were analysed descriptively. The open questions were analysed thematically.

### Ethical approval

Advice was sought from the local research ethics committee, which covered all of the hospital sites involved, who considered that this project did not require ethical review by a NHS Research Ethics Committee. Prior to any observation of a drug manipulation verbal consent from the nurses involved was sought. The protocol specified that if a potential drug error was witnessed during the observation then the researchers observing the drug manipulation would intervene and request that the calculation or measurement was checked. If the error was not corrected then the observer would then make the nurse involved aware of the error prior to any drug administration to a patient.

### Estimating the requirement for manipulation

The observational study was a scoping study which aimed to examine examples of the type and nature of drug manipulations across neonatal and paediatric inpatient areas. In the absence of an evidence-base for this practice, the sample frame was designed to sample maximum variation by observing as many different types of drug manipulation as possible. This led to targeted observations, or purposive sampling. The resources available could only capture a diverse sample of manipulations if the research staff were deployed flexibly. That is, the research team could either capture ad hoc manipulations they had not yet observed, or, they could capture a consistent sample of predictable manipulations. Accordingly the study was not designed to estimate the frequency of each manipulation. As a supplement to this study, prescription data was collected for five days for all neonatal and paediatric inpatients in the 21 different inpatient areas used in the observational study. These data were reviewed by an experienced paediatric pharmacist who used them to provide an estimate of the requirement for manipulation [14]. This study estimated that 10% of the evaluated drug administration episodes would require a manipulation [14]. Given the nature and wide geographical distribution of the questionnaire study we were unable to ask respondents to assess the frequency of the manipulations.

## Results

### Observational study

**Figure 1:**
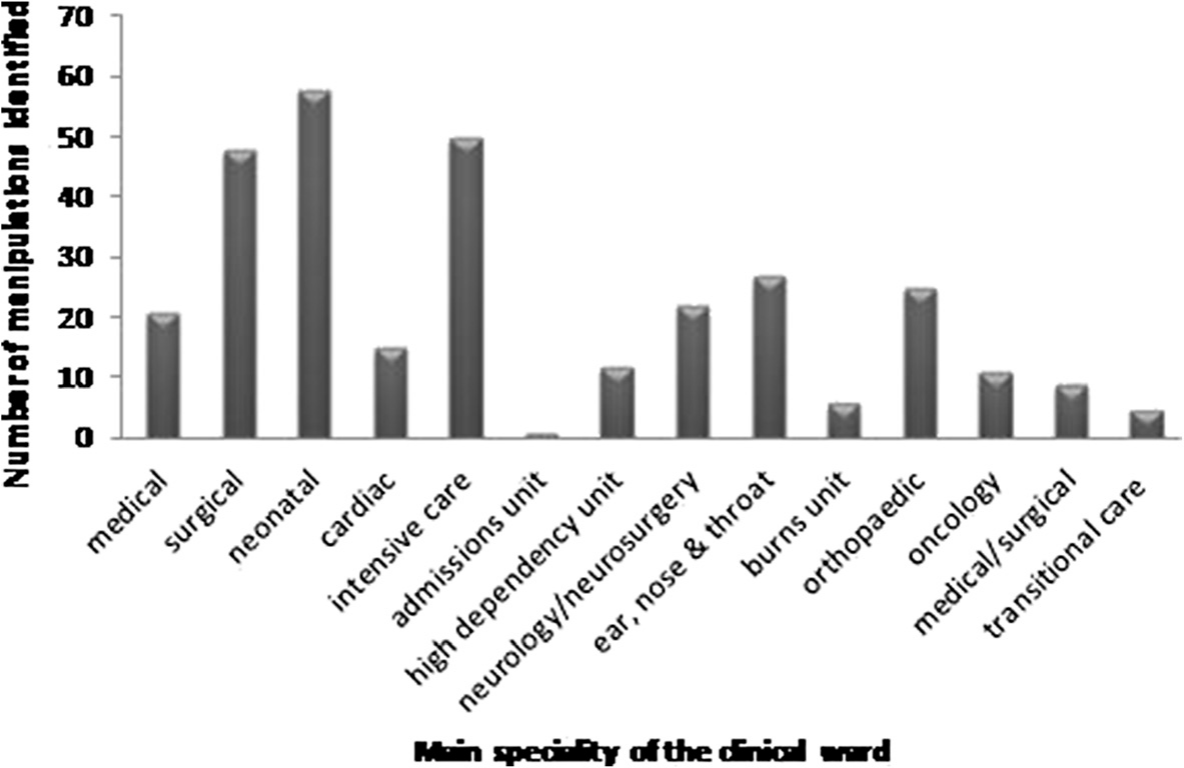
Clinical areas where manipulations were identified during the observational study.

During the observational study, 310 manipulations were identified. Researchers did not observe or have to prevent any drug errors during this study. Many manipulations were identified in specialist areas or clinical areas with more highly dependent patients, though manipulations were identified in all types of wards. Figure 1 details the clinical areas where manipulations were identified during the observational study. Of the 310 identified manipulations, 54 (17%) were observed. The patients observed receiving manipulated drugs encompassed those with a wide variety of conditions and included an age range from 2 days to 19 years. The main reasons for non-observation were: the patient not receiving the drug at the prescribed time (changes in the patient’s condition, patient in theatre); patient discharged from the ward; changes to the patient’s prescription (the changes in dose or drug meant that a manipulation may no longer be required) and difficulties with trying to anticipate when ‘as required’ drugs would be needed.

Tables 2 and 3 detail the dosage forms and different drugs involved in the manipulations reported in the observational study.

**Table 2:** Dosage forms of the drug manipulations identified in the observational study and questionnaire

**Dosage forms manipulated**	**Number of manipulations identified – observational study (percentage)**	**Number of manipulations identified – questionnaire (percentage)**	**Number of different drugs involved – observational study (percentage)**	**Number of different drugs involved – questionnaire (percentage)**
**Tablet**	191 (61.6%)	86 (45.7%)	28 (48.3%)	30 (50.8%)
**Intravenous injection**	65 (21.0%)	22 (11.7%)	18 (31.0%)	13 (21.3%)
**Sachet**	30 (9.7%)	2 (1.1%)	4 (6.9%)	1 (1.6%)
**Transdermal patch**	10 (3.2%)	20 (10.6%)	1 (1.7%)	2 (3.3%)
**Suppository**	6 (1.9%)	15 (8.0%)	3 (5.2%)	4 (6.6%)
**Capsule**	4 (1.3%)	15 (8.0%)	3 (5.2%)	8 (13.1%)
**Nebuliser solution**	4 (1.3%)	22 (11.7%)	1 (1.7%)	1 (1.6%)
**Enema**	0 (0%)	6 (3.2%)	0 (0%)	1 (1.6%)
**Total**	310 (100%)	188 (100%)	58 (100%)	60 (100%)

**Table 3:** **Drugs identified as manipulated using British National Formulary for Children (BNFC) classification**[19]

**BNFC classification**	**Number of manipulations identified – observational study (percentage)**	**Number of manipulations identified – questionnaire (percentage)**
**Analgesic**	92 (29.7%)	33 (17.6%)
**Proton pump inhibitor**	24 (7.7%)	24 (12.8%)
**Antimuscarinic**	18 (5.4%)	20 (10.6%)
**Antiemetic**	17 (5.5%)	5 (2.7%)
**Alginate preparation**	16 (5.2%)	2 (1.1%)
**Antiplatelet**	15 (4.8%)	3 (1.6%)
**Opioid analgesic**	14 (4.5%)	2 (1.1%)
**Benzodiazepines**	13 (4.2%)	2 (1.1%)
**Antiepileptic**	12 (3.9%)	6 (3.2%)
**Antibiotic**	11 (3.5%)	7 (3.7%)
**Neuromuscular blocking**	11 (3.5%)	0
**Steroid**	10 (3.2%)	12 (6.4%)
**ACE inhibitor**	5 (1.6%)	2 (1.1%)
**Bronchodilator**	5 (1.6%)	23 (12.2%)
**Minerals**	5 (1.6%)	2 (1.1%)
**Thyroid hormone**	5 (1.6%)	1 (0.5%)
**Vasodilator**	5 (1.6%)	1 (0.5%)
**Diuretic**	4 (1.3%)	0
**Drugs affecting the ductus arteriosus**	3 (1.0%)	0
**Insulin**	3 (1.0%)	0
**Laxative**	3 (1.0%)	4 (2.1%)
**Antipsychotic**	2 (0.6%)	1 (0.5%)
**Antiviral**	2 (0.6%)	1 (0.5%)
**Flu prophylaxis**	2 (0.6%)	0
**Hypothalamic & pituitary hormone**	2 (0.6%)	0
**H2 antagonist**	2 (0.6%)	8 (4.3%)
**Inotrope**	2 (0.6%)	0
**Anticoagulant**	1 (0.3%)	2 (1.1%)
**Antidepressant**	1 (0.3%)	0
**Antihypertensive**	1 (0.3%)	1 (0.5%)
**Antimotility**	1 (0.3%)	3 (1.6%)
**Pineal hormone**	1 (0.3%)	2 (1.1%)
**Smooth muscle relaxant**	1 (0.3%)	0
**Osmotic laxative**	0	6 (3.2%)
**Immunosuppressant**	0	4 (2.1%)
**Sedation**	0	3 (1.6%)
**Calcium channel blocker**	0	2 (1.1%)
**Cytotoxic**	0	2 (1.1%)
**Vitamin**	0	2 (1.1%)
**Beta-blocker**	0	1 (0.5%)
**Skeletal muscle relaxant**	0	1 (0.5%)
**Not in BNFC**	1 (0.3%)	0
**Total**	310 (100%)	188 (100%)

Of the 40 tablet manipulations 25 (62.5%) of the tablets were cut, 12 (30%) dispersed, one (2.5%) crushed, one (2.5%) was broken by hand and there was one (2.5%) manipulation where the tablet was split in half first and then dispersed so that a quarter of the dosage form prior to manipulation could be withdrawn and administered. In three (11.5%) of the 25 tablet manipulations where the tablet had been split the manipulation had to be repeated. In two cases the tablet crumbled while being split, whilst in the third case the tablet split unevenly. During a further nine (34.6%) of these manipulations there was also visible powder generated when the tablet was split.

**Figure 2:**
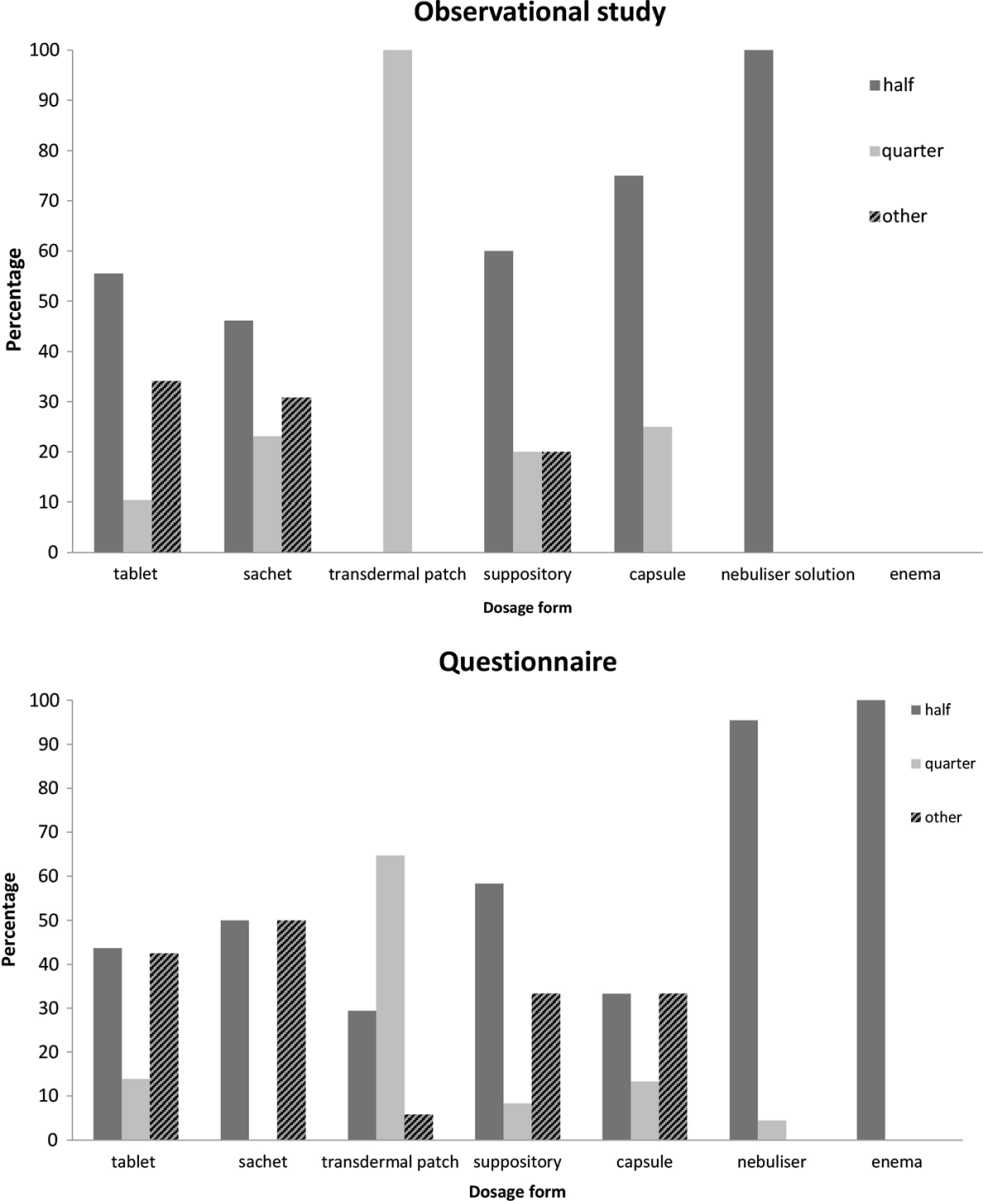
Percentage of the prescribed dose required of the available dose for solid dosage forms found in the observational study and the questionnaire.

The predominant proportions required for non-intravenous administration were either a half or a quarter/three quarters of the dosage form prior to manipulation, though other proportions were also required (Figure 2). The intravenous drug manipulations were all reported in specialist areas; 60% in the specialist neonatal unit, 1.5% in the cardiac unit and 38.5% in paediatric intensive care.

### Questionnaire study

**Figure 3:**
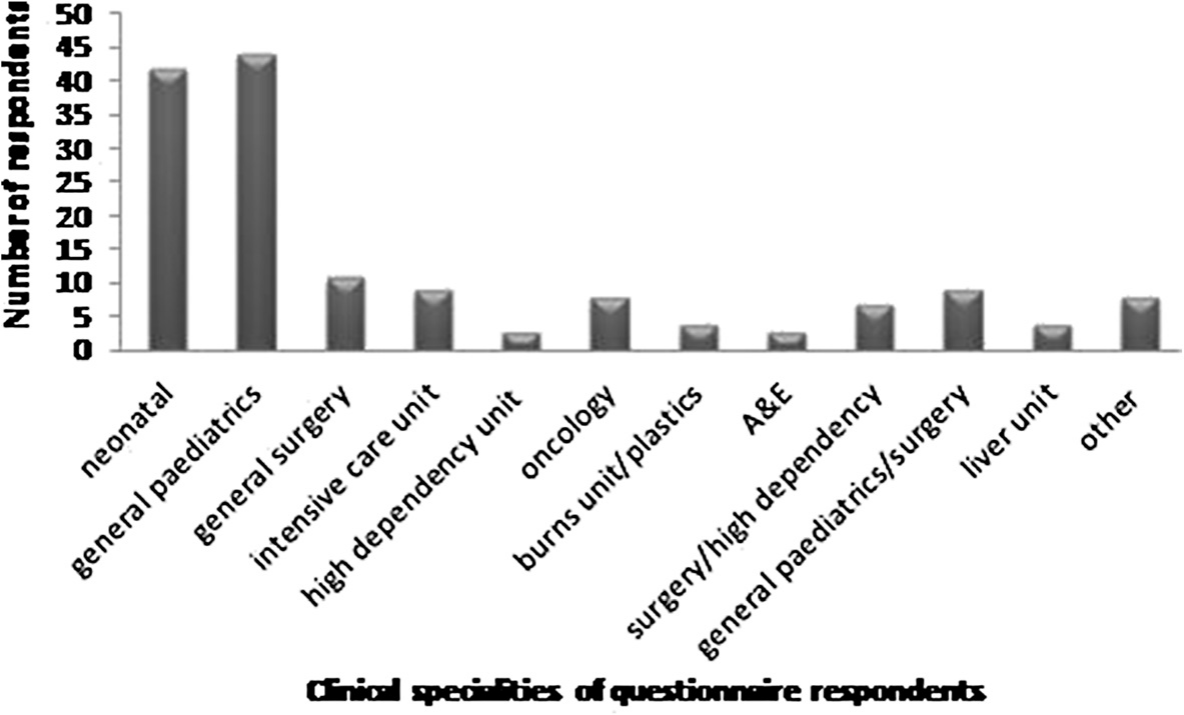
Clinical areas questionnaire respondents currently work in.

560 questionnaires were distributed with 153 returned (27.3% response rate). Questionnaire respondents worked in a variety of different specialties of neonatal and paediatric practice (Figure 3). 258 potential drug manipulations were reported by the questionnaire respondents. On review 70 of these either did not meet the criteria to be considered manipulations or had not reported sufficient data, leaving 188 (73%) to be included.

The predominant proportions required for non-intravenous administration were either a half or a quarter/three quarters of the dosage form prior to manipulation, although other proportions were required (Figure 2). The intravenous drug manipulations were reported predominantly in neonatal areas (68.2%), with 18.2% in general paediatrics, 4.5% in each of intensive care, accident and emergency and surgery/high dependency.

For 55% of the reported manipulations the sole reason for the manipulation was that there was ‘no suitable preparation or strength available’. Further reasons given for manipulations were, patient preference (reported for 13 (6.9%) manipulations) and usual practice 23 (12.2%).

Thirty five percent of respondents to the questionnaire reported concerns with the accuracy of the dose achieved following manipulation. Respondents also noted the importance of good communication between health care professionals and the need for availability of clear drug preparation and administration protocols and/or policies for such scenarios.

## Discussion

This study used two complementary approaches to investigate the scope of manipulations of drugs to achieve the prescribed dose in neonatal and paediatric practice. The results provide an overview of the issues that arise when manipulations are undertaken. Although manipulations were reported more frequently from more specialist areas, such as neonatal and paediatric intensive care areas, they occurred in all paediatric in-patient areas. The manipulations identified involved a wide range of drugs.

Patient preference was the sole reason for 15% of the manipulations reported in the questionnaire. This was also found during the observational study. For example one child preferred to take half a tablet even though a liquid formulation was available because taking the liquid involved a large volume of a liquid whose taste they did not like.

The predominant concern noted by questionnaire respondents was whether the manipulated medicine would provide an accurate dose. These concerns have also been described previously with halving or quartering tablets [8,15-17]. The need for caution when splitting tablets with short half-lives or low therapeutic indices [17] has been highlighted.

A novel finding here is that sizable number of reported and observed manipulations did not correspond to half or quarter of the dosage form. The accuracy of dividing tablets into other fractions is even more unclear that halves or quarters. The potential lack of accuracy during manipulations implied by a prescription may mean that the actual dose delivered to the patient is not known. It seems likely that prescribers are often unaware of the dosage form (and strength/concentration) that will be used to administer the dose required or the potential for inaccuracy that arises from their prescribed doses.

This study also found that tablets are manipulated by dispersion in liquid and measurement of a proportion. Highly variable dosing may occur when insoluble drugs are dispersed in water [7]. Dispersible tablets can also yield inconsistent doses when withdrawn from different depths of the container [18].

Manipulations with the aim of achieving the required dose are also undertaken with capsules, sachets, suppositories, nebulisers, enemas and transdermal patches. The manipulation of these other dosage forms has not been previously investigated. The adverse effects associated with administering manipulated drugs, of any dosage form, are unknown. There is a need to conduct research about high impact manipulations, such as those that involve an active ingredient with a narrow therapeutic index or where the physicochemical properties of the active ingredient may lead to significant changes in bioavailability following a manipulation.

This study was designed to scope the nature and occurrence of manipulations and identify priorities for further research. As such, we used a purposive sampling approach for maximum variability for the observational study and a sample of convenience for the questionnaire study. This precludes quantitative generalisation of our results. The supplementary quantitative study estimated that manipulations would be required in approximately 10% of drug administration episodes in the clinical areas studied in the observational study. Nevertheless, our methods were sufficiently fine-grained and pragmatic to yield data that indicate the need for action. The dose of commonly used medicines was usually calculated from body weight and dose/kg according to standard practice [19] without rounding to take account of dosage form/strength. Manipulation of the dosage form was undertaken with the aim of administering the precise dose. If other centres are using standard sources, such as BNFC, they are also likely to be conducting manipulations. The nature of the issues we have identified would not change if data were collected from more centres. We note that our methods gave complementary results. Any further research on the nature of the problem will need approaches that provide more than one perspective.

Our results demonstrate that manipulations of dosage forms are integral to paediatric practice. However, it is not entirely clear who is responsible for manipulations. There are two relevant situations, firstly, when the manipulation is conducted because a suitable dosage form is not available. This situation may arise because there is no suitable dosage form on the market, because a purchasing decision has prevented a suitable dosage form from being issued from Pharmacy or because a suitable dosage form is not available on the ward at the point of need (e.g. at night when Pharmacy staff may not be available). Secondly, the nurse makes a professional judgment to meet a patient’s preference when a dosage form is available. We have developed generic guidelines about the manipulation of dosage forms in children (available from the authors). We suggest that paediatric units should have policies and procedures to cover manipulation of dosage forms. This should ensure that there is corporate responsibility for manipulations. For example, purchasing decisions need to take account of the manipulations that may result from the choice of available products. Nurses should have access to the relevant information and the support from their institution to make professional judgments about manipulations, many of which are done on off-label or unlicensed medicines.

## Conclusion

In conclusion manipulations are intrinsic to contemporary paediatrics. The manipulations conducted in the UK are not supported by relevant data and raise important issues of safety, policy and practice. Our data indicates priorities for policy and research in a neglected but important area of medicines management.

## Competing interests

The authors declare that they have no competing interests.

## Authors’ contributions

AN, RR, US, CB, JC, JF MP and MT conceived and designed the study. RR organised and collected the data. RR, AN and MT prepared the article. All authors contributed to critical revision of the article and the final draft. All authors read and approved the final manuscript.

## Pre-publication history

The pre-publication history for this paper can be accessed here:

http://www.biomedcentral.com/1471-2431/13/81/prepub
